# A single parameter method for secure privacy preserving record linkage

**DOI:** 10.23889/ijpds.v9i5.2531

**Published:** 2024-09-18

**Authors:** Peter Christen, Sumayya Ziyad, Anushka Vidanage, Charini Nanayakkara, Rainer Schnell

**Affiliations:** 1 Australian National University; 2 University Duisburg-Essen

## Objectives

Data linkage is the process of matching records that refer to the same entities (often people) across databases. In applications such as health research or government services, the databases to be linked are often sensitive and cannot be shared between organisations. Privacy-preserving record linkage (PPRL) aims to overcome this challenge by facilitating the comparison of encoded or encrypted records without having to share sensitive data. Most existing PPRL techniques are based on heuristics and they have limitations in the privacy protection they offer, such as being vulnerable to certain cryptanalysis attacks. Furthermore, existing PPRL methods have multiple parameters, which, if not set properly by the user, can result in sub-optimal linkage quality and reduced privacy protection.

## Approach

We present a novel PPRL method that uses random reference q-gram sets to generate bit-arrays that represent sensitive values. Our method has a single parameter to be set by the user that trades scalability with linkage quality and privacy protection. All other parameters are either data-driven or have strong bounds based on this user parameter.

## Results

We conceptually analyse our method and conduct experiments on multiple databases. The results demonstrate that our method provides high linkage quality and strong privacy protection while being scalable to link very large databases.

## Conclusion

Our novel PPRL method provides high linkage quality, scalability, and improved privacy protection compared to existing PPRL methods such as Bloom filter encoding. A major advantage of our method is that it requires a single parameter to be set by the user.

